# Trithia­cyanuric acid: a second triclinic polymorph

**DOI:** 10.1107/S1600536810033234

**Published:** 2010-08-21

**Authors:** Iván Brito, Joselyn Albanez, Michael Bolte

**Affiliations:** a Departamento de Química, Facultad de Ciencias Básicas, Universidad de Antofagasta, Casilla 170, Antofagasta, Chile; b Institut für Anorganische Chemie der Goethe-Universität Frankfurt, Max-von-Laue-Strasse 7, D-60438 Frankfurt am Main, Germany

## Abstract

The title compound, C_3_H_3_N_3_S_3_, is a triclinic modification. The other reported modification crystallizes with just one mol­ecule in the asymmetric unit, [Guo *et al.* (2006 ▶). *Cryst. Growth Des.* **6**, 846–848] and was solved by power X-ray diffraction data. The present modification has *Z*′ = 2. In the crystal, mol­ecules are linked by strong intra­molecular N—H⋯S hydrogen bonds with set graph-motif *R*_2_^2^(8). In both mol­ecules, all of the N atoms and two of the S atoms are involved in hydrogen bonding, with an average H⋯S distance of 2.58 Å and N—H⋯S angles in the range 163–167°. π–π stacking inter­actions are not observed. In the solid state, the mol­ecules exist in the thione form. The mol­ecular and supra­molecular properties are similar in both polymorphs.

## Related literature

For general background to trithia­cyanuric acid, see: Henke *et al.*, (2000 ▶); Iltzsch & Tankersley (1993 ▶, 1994 ▶); Clegg *et al.* (1998 ▶); Yamanari *et al.* (1993 ▶); Bailey *et al.* (2001 ▶); Hunks *et al.* (1999 ▶); Tzeng *et al.* (1997 ▶). For hydrogen-bond motifs, see: Bernstein *et al.* (1995 ▶). For the other triclinic polymorph of trithia­cyanuric acid, see: Guo *et al.* (2006 ▶). For the biological properties of trithia­cyanuric acid, see: Iltzsch & Tankersley (1993 ▶, 1994 ▶).
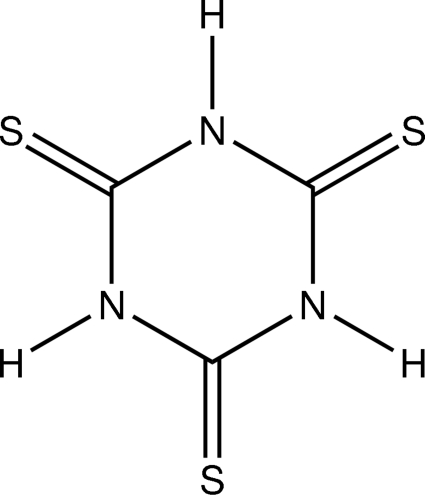


## Experimental

### 

#### Crystal data

C_3_H_3_N_3_S_3_
*M* *_r_* = 177.26
Triclinic, 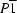

*a* = 6.9690 (11) Å
*b* = 8.807 (1) Å
*c* = 11.3557 (16) Å
α = 78.96 (1)°
β = 75.072 (12)°
γ = 77.234 (11)°
*V* = 650.07 (16) Å^3^
*Z* = 4
Mo *K*α radiation
μ = 1.04 mm^−1^
*T* = 173 K
0.27 × 0.25 × 0.22 mm

#### Data collection

Stoe IPDS II two-circle diffractometer
Absorption correction: multi-scan (*MULABS*; Spek, 2003 ▶; Blessing, 1995 ▶) *T*_min_ = 0.766, *T*_max_ = 0.803
5659 measured reflections
2292 independent reflections
1344 reflections with *I* > 2σ(*I*)
*R*_int_ = 0.111

#### Refinement

*R*[*F* ^2^ > 2σ(*F* ^2^)] = 0.077
*wR*(*F* ^2^) = 0.210
*S* = 0.91
2292 reflections
163 parameters
H-atom parameters constrained
Δρ_max_ = 0.81 e Å^−3^
Δρ_min_ = −0.51 e Å^−3^

### 

Data collection: *X-AREA* (Stoe & Cie, 2001 ▶); cell refinement: *X-AREA*; data reduction: *X-AREA*; program(s) used to solve structure: *SHELXS97* (Sheldrick, 2008 ▶); program(s) used to refine structure: *SHELXL97* (Sheldrick, 2008 ▶); molecular graphics: *XP* (Sheldrick, 2008 ▶); software used to prepare material for publication: *SHELXL97*.

## Supplementary Material

Crystal structure: contains datablocks I, global. DOI: 10.1107/S1600536810033234/fl2313sup1.cif

Structure factors: contains datablocks I. DOI: 10.1107/S1600536810033234/fl2313Isup2.hkl

Additional supplementary materials:  crystallographic information; 3D view; checkCIF report

## Figures and Tables

**Table 1:** Hydrogen-bond geometry (Å, °)

*D*—H⋯*A*	*D*—H	H⋯*A*	*D*⋯*A*	*D*—H⋯*A*
N2—H2⋯S1*A*^i^	0.88	2.53	3.383 (7)	163
N4—H4⋯S5*A*^ii^	0.88	2.62	3.473 (6)	165
N6—H6⋯S5*A*	0.88	2.62	3.480 (6)	166
N2*A*—H2*A*⋯S1^iii^	0.88	2.48	3.342 (7)	167
N4*A*—H4*A*⋯S5^iv^	0.88	2.64	3.500 (6)	167
N6*A*—H6*A*⋯S5	0.88	2.61	3.476 (6)	167

Symmetry codes: (i) 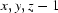; (ii) 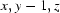; (iii) 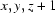; (iv) 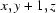.

**Fig. 1.**
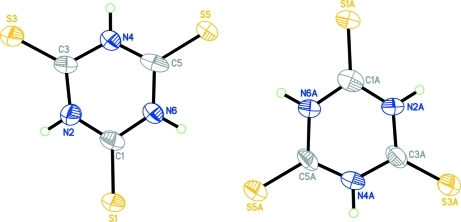
The two molecules in the asymmetric unit of the title compound with displacement ellipsoids at the 50% probability level.

**Fig. 2.**
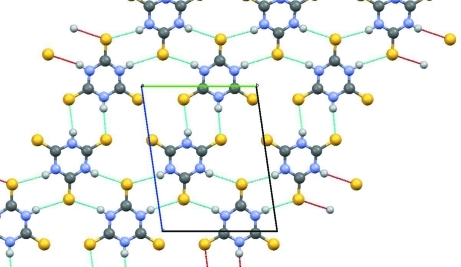
Crystal structure of (I) showing a single sheet viewed along [010] direction.

## supplementary crystallographic information

### Comment

Trithiocyanuric acid and its trisodium salt are widely applied in industry, analytical chemistry and biochemistry. For example its trisodium salt is used as a precipitating agent for many heavy metals from contaminated water (Henke *et al.*, 2000). Moreover, it was found that the acid inhibits the Toxoplasma gondii uracil phosphoribosyltransferase enzyme *in vitro* better than 5-fluorouracil and emimcin compounds showing an antitoxoplasmal activity (Iltzsch *et al.*, 1993, 1994). The title compound bearing three N,*S* donor sets can display a great versatility of coordination As a matter of fact it can use from one to all the six of its donor atoms (Clegg *et al.*, 1998; Yamanari *et al.*, 1993) to form polynuclear complexes (Bailey *et al.*, 2001; Hunks *et al.*, 1999). Its capability to act as a bridging ligand is also shown in polymeric compounds (Tzeng *et al.*, 1997). The structure of compound (II) (Guo *et al.*, 2006) was solved by powder X-ray diffraction using the direct-space genetic algorithm technique for structure solution followed by Rietveldt refinement. The authors were unable to obtain single-crystals due to the title compound having a strong propensity to form co-crystals (solvates) in crystallization experiments from the types of solvents in which it is readily soluble. They reported that their modification crystallized with just one molecule in the asymmetric unit, (*Z*=2) from density considerations.

We are particularly interested in the utility of the title compound due its great versatility for the fabrication of different coordination polymers. We report here the structure of a new polymorph of (I) isolated during attempts to synthetize coordination polymers between (I) and PdCl_2, _Fig 1. The present modification has *Z*'=2. The bond lengths C— S and C— N are 1.658 (7)Å and 1.355 (9) Å. The two molecules in the asymmetric unit are linked by two strong N—H···S intramolecular hydrogen bonds with set graph-motif *R*^2^_2_(8) (Bernstein *et al.*, 1995), Fig 2. In both molecules of the asymmetric unit all of the nitrogen atoms and two of the sulfur atoms are involved in hydrogen bonding with an average H— S distance of 2.58 Å and N— H— S angle ranging from 163–167° (Table 1). π-π stacking interactions were not observed. In the solid state the title compound exists in the thione form.

The common feature of both polymorphs is that the crystal structure comprises sheets of molecules. In (I) these sheets are parallel to (100) and in (II) parallel to (1–20) planes (consistent with the fact that the PXRD pattern has a peak of dominant intesity, indexed as (1–20)). Within the sheets, there is extensive N—H···S hydrogen bonding. Each N—H bond is a donor in one N—H···S hydrogen bond, but the S atoms in the molecule differ in their behavior as hydrogen bond acceptors. Thus, one S atom (in both molecules of the aymmetric unit of (I)) accepts two N—H···S hydrogen bonds, one S atom accepts one N—H···S hydrogen bond, and the other S atom is not involved in any hydrogen bonding (Table 1). In the hydrogen bonding network groups of six molecules are arranged in a cyclic manner, at the center of which four S atoms (including two S atoms not involved in hydrogen bonding) are in van der Waals contact (S···S 3.40–3.90Å for (I) and 3.37–3.52Å for (II)).In general the molecular and supramolecular properties are  similar in both polymorphs.

### Experimental

A solution containing 1:1 molar ratio of PdCl_2_ (0.2 mmol, 35.6 mg) and trihiocyanuric acid (0.2 mmol, 35.5 mg) in acetonitrile/chloroform (1:1) was stirred at room temperature for 30 min, and the mixture was filtered. Yellow single crystals suitable for X-ray investigation were obtained from above filtrate by slow evaporation of the solution. FT—IR (KBr, pellets, cm^-1^): ν(C— N)1530 s, 1358*m*, 1117 s; ν(C— S) 785w, 744w; ν(N— H) 3492w.

### Refinement

All H atoms were placed in idealized positions with d(N—H) = 0.88Å and refined using a riding model with *U*_iso_(H) fixed at 1.2 *U*_eq_(N).

### Crystal data

**Table:**

C_3_H_3_N_3_S_3_	*Z* = 4
*M**_r_* = 177.26	*F*(000) = 360
Triclinic, *P*1	*D*_x_ = 1.811 Mg m^−^^3^
Hall symbol: -P 1	Mo *K*α radiation, λ = 0.71073 Å
*a* = 6.9690 (11) Å	Cell parameters from 3753 reflections
*b* = 8.807 (1) Å	θ = 3.5–25.8°
*c* = 11.3557 (16) Å	µ = 1.04 mm^−^^1^
α = 78.96 (1)°	*T* = 173 K
β = 75.072 (12)°	Block, light yellow
γ = 77.234 (11)°	0.27 × 0.25 × 0.22 mm
*V* = 650.07 (16) Å^3^	

### Data collection

**Table:**

Stoe IPDS II two-circle diffractometer	2292 independent reflections
Radiation source: fine-focus sealed tube	1344 reflections with *I* > 2σ(*I*)
graphite	*R*_int_ = 0.111
ω scans	θ_max_ = 25.0°, θ_min_ = 3.5°
Absorption correction: multi-scan (*MULABS*; Spek, 2003; Blessing, 1995)	*h* = −8→8
*T*_min_ = 0.766, *T*_max_ = 0.803	*k* = −10→10
5659 measured reflections	*l* = −13→13

### Refinement

**Table:**

Refinement on *F*^2^	Primary atom site location: structure-invariant direct methods
Least-squares matrix: full	Secondary atom site location: difference Fourier map
*R*[*F*^2^ > 2σ(*F*^2^)] = 0.077	Hydrogen site location: inferred from neighbouring sites
*wR*(*F*^2^) = 0.210	H-atom parameters constrained
*S* = 0.91	*w* = 1/[σ^2^(*F*_o_^2^) + (0.1165*P*)^2^] where *P* = (*F*_o_^2^ + 2*F*_c_^2^)/3
2292 reflections	(Δ/σ)_max_ < 0.001
163 parameters	Δρ_max_ = 0.81 e Å^−^^3^
0 restraints	Δρ_min_ = −0.51 e Å^−^^3^

### Special details

**Table:**

Geometry. All e.s.d.'s (except the e.s.d. in the dihedral angle between two l.s. planes) are estimated using the full covariance matrix. The cell e.s.d.'s are taken into account individually in the estimation of e.s.d.'s in distances, angles and torsion angles; correlations between e.s.d.'s in cell parameters are only used when they are defined by crystal symmetry. An approximate (isotropic) treatment of cell e.s.d.'s is used for estimating e.s.d.'s involving l.s. planes.
Refinement. Refinement of *F*^2^ against ALL reflections. The weighted *R*-factor *wR* and goodness of fit *S* are based on *F*^2^, conventional *R*-factors *R* are based on *F*, with *F* set to zero for negative *F*^2^. The threshold expression of *F*^2^ > σ(*F*^2^) is used only for calculating *R*-factors(gt) *etc*. and is not relevant to the choice of reflections for refinement. *R*-factors based on *F*^2^ are statistically about twice as large as those based on *F*, and *R*- factors based on ALL data will be even larger.

### Fractional atomic coordinates and isotropic or equivalent isotropic displacement parameters (Å^2^)

**Table:**

	*x*	*y*	*z*	*U*_iso_*/*U*_eq_	
S1	0.2566 (3)	0.6245 (2)	0.36799 (18)	0.0446 (5)	
S3	0.2538 (3)	0.0251 (2)	0.37171 (18)	0.0517 (6)	
S5	0.2656 (3)	0.2258 (2)	0.77989 (16)	0.0442 (5)	
C1	0.2473 (10)	0.4435 (8)	0.4452 (7)	0.0387 (17)	
N2	0.2307 (9)	0.3231 (6)	0.3934 (6)	0.0407 (14)	
H2	0.2101	0.3456	0.3183	0.049*	
C3	0.2428 (11)	0.1690 (8)	0.4463 (7)	0.0399 (17)	
N4	0.2461 (9)	0.1472 (7)	0.5705 (5)	0.0439 (15)	
H4	0.2420	0.0523	0.6120	0.053*	
C5	0.2551 (11)	0.2619 (8)	0.6317 (7)	0.0452 (19)	
N6	0.2608 (9)	0.4071 (7)	0.5642 (5)	0.0399 (14)	
H6	0.2742	0.4825	0.6007	0.048*	
S1A	0.2459 (3)	0.3512 (2)	1.08995 (17)	0.0444 (5)	
S3A	0.2355 (4)	0.9528 (2)	1.0898 (2)	0.0534 (6)	
S5A	0.2560 (3)	0.7492 (2)	0.67644 (18)	0.0448 (5)	
C1A	0.2520 (11)	0.5327 (9)	1.0160 (7)	0.0454 (19)	
N2A	0.2401 (9)	0.6582 (6)	1.0724 (6)	0.0410 (14)	
H2A	0.2276	0.6413	1.1527	0.049*	
C3A	0.2460 (11)	0.8090 (8)	1.0143 (7)	0.0416 (17)	
N4A	0.2538 (9)	0.8283 (7)	0.8917 (6)	0.0426 (15)	
H4A	0.2531	0.9246	0.8521	0.051*	
C5A	0.2627 (11)	0.7150 (8)	0.8237 (7)	0.0406 (17)	
N6A	0.2661 (9)	0.5667 (6)	0.8916 (6)	0.0422 (14)	
H6A	0.2783	0.4876	0.8515	0.051*	

### Atomic displacement parameters (Å^2^)

**Table:**

	*U*^11^	*U*^22^	*U*^33^	*U*^12^	*U*^13^	*U*^23^
S1	0.0670 (12)	0.0315 (9)	0.0349 (10)	−0.0101 (8)	−0.0176 (8)	0.0060 (7)
S3	0.0823 (14)	0.0341 (10)	0.0403 (11)	−0.0080 (9)	−0.0232 (10)	0.0003 (9)
S5	0.0698 (13)	0.0347 (9)	0.0289 (10)	−0.0112 (8)	−0.0175 (9)	0.0039 (8)
C1	0.041 (4)	0.037 (4)	0.036 (4)	−0.006 (3)	−0.016 (3)	0.008 (3)
N2	0.055 (4)	0.033 (3)	0.037 (3)	−0.007 (3)	−0.018 (3)	−0.005 (3)
C3	0.053 (4)	0.027 (3)	0.036 (4)	−0.003 (3)	−0.013 (3)	0.004 (3)
N4	0.068 (4)	0.034 (3)	0.033 (3)	−0.009 (3)	−0.021 (3)	0.000 (3)
C5	0.046 (4)	0.040 (4)	0.040 (4)	−0.007 (3)	−0.011 (3)	0.017 (3)
N6	0.055 (4)	0.037 (3)	0.031 (3)	−0.009 (3)	−0.018 (3)	0.001 (3)
S1A	0.0619 (12)	0.0318 (9)	0.0384 (11)	−0.0096 (8)	−0.0169 (8)	0.0069 (8)
S3A	0.0864 (15)	0.0364 (10)	0.0400 (11)	−0.0104 (9)	−0.0230 (10)	−0.0008 (8)
S5A	0.0616 (12)	0.0381 (10)	0.0333 (10)	−0.0080 (8)	−0.0162 (8)	0.0047 (8)
C1A	0.045 (4)	0.046 (4)	0.041 (4)	−0.008 (3)	−0.008 (3)	0.004 (4)
N2A	0.061 (4)	0.025 (3)	0.037 (3)	−0.010 (2)	−0.015 (3)	0.003 (3)
C3A	0.053 (4)	0.044 (4)	0.026 (4)	−0.005 (3)	−0.013 (3)	0.001 (3)
N4A	0.058 (4)	0.032 (3)	0.036 (3)	−0.008 (3)	−0.018 (3)	0.008 (3)
C5A	0.052 (4)	0.043 (4)	0.027 (4)	−0.003 (3)	−0.021 (3)	0.004 (3)
N6A	0.063 (4)	0.027 (3)	0.037 (3)	−0.007 (3)	−0.018 (3)	0.003 (3)

### Geometric parameters (Å, °)

**Table:**

S1—C1	1.672 (6)	S1A—C1A	1.662 (7)
S3—C3	1.632 (8)	S3A—C3A	1.639 (8)
S5—C5	1.669 (8)	S5A—C5A	1.652 (7)
C1—N2	1.346 (9)	C1A—N2A	1.357 (10)
C1—N6	1.350 (9)	C1A—N6A	1.369 (10)
N2—C3	1.368 (8)	N2A—C3A	1.370 (8)
N2—H2	0.8800	N2A—H2A	0.8800
C3—N4	1.392 (9)	C3A—N4A	1.359 (9)
N4—C5	1.352 (10)	N4A—C5A	1.356 (10)
N4—H4	0.8800	N4A—H4A	0.8800
C5—N6	1.362 (8)	C5A—N6A	1.382 (9)
N6—H6	0.8800	N6A—H6A	0.8800
			
N2—C1—N6	115.1 (6)	N2A—C1A—N6A	114.9 (6)
N2—C1—S1	122.9 (5)	N2A—C1A—S1A	123.6 (6)
N6—C1—S1	122.0 (6)	N6A—C1A—S1A	121.5 (6)
C1—N2—C3	126.4 (6)	C1A—N2A—C3A	125.2 (6)
C1—N2—H2	116.8	C1A—N2A—H2A	117.4
C3—N2—H2	116.8	C3A—N2A—H2A	117.4
N2—C3—N4	113.0 (6)	N4A—C3A—N2A	114.2 (7)
N2—C3—S3	123.7 (6)	N4A—C3A—S3A	123.9 (6)
N4—C3—S3	123.3 (5)	N2A—C3A—S3A	121.8 (6)
C5—N4—C3	124.5 (6)	C5A—N4A—C3A	127.0 (6)
C5—N4—H4	117.8	C5A—N4A—H4A	116.5
C3—N4—H4	117.8	C3A—N4A—H4A	116.5
N4—C5—N6	115.9 (7)	N4A—C5A—N6A	113.3 (6)
N4—C5—S5	121.8 (5)	N4A—C5A—S5A	124.2 (5)
N6—C5—S5	122.3 (7)	N6A—C5A—S5A	122.4 (6)
C1—N6—C5	124.7 (7)	C1A—N6A—C5A	125.4 (7)
C1—N6—H6	117.7	C1A—N6A—H6A	117.3
C5—N6—H6	117.7	C5A—N6A—H6A	117.3
			
N6—C1—N2—C3	−5.6 (10)	N6A—C1A—N2A—C3A	−1.6 (10)
S1—C1—N2—C3	172.9 (6)	S1A—C1A—N2A—C3A	179.6 (6)
C1—N2—C3—N4	8.8 (10)	C1A—N2A—C3A—N4A	3.6 (10)
C1—N2—C3—S3	−170.8 (6)	C1A—N2A—C3A—S3A	−178.6 (6)
N2—C3—N4—C5	−5.7 (10)	N2A—C3A—N4A—C5A	−2.3 (11)
S3—C3—N4—C5	173.9 (6)	S3A—C3A—N4A—C5A	−180.0 (6)
C3—N4—C5—N6	0.1 (10)	C3A—N4A—C5A—N6A	−0.8 (10)
C3—N4—C5—S5	−178.1 (6)	C3A—N4A—C5A—S5A	175.6 (6)
N2—C1—N6—C5	−1.2 (10)	N2A—C1A—N6A—C5A	−2.1 (11)
S1—C1—N6—C5	−179.7 (6)	S1A—C1A—N6A—C5A	176.8 (6)
N4—C5—N6—C1	3.7 (10)	N4A—C5A—N6A—C1A	3.2 (10)
S5—C5—N6—C1	−178.1 (5)	S5A—C5A—N6A—C1A	−173.3 (6)

### Hydrogen-bond geometry (Å, °)

**Table:**

*D*—H···*A*	*D*—H	H···*A*	*D*···*A*	*D*—H···*A*
N2—H2···S1A^i^	0.88	2.53	3.383 (7)	163.
N4—H4···S5A^ii^	0.88	2.62	3.473 (6)	165.
N6—H6···S5A	0.88	2.62	3.480 (6)	166.
N2A—H2A···S1^iii^	0.88	2.48	3.342 (7)	167.
N4A—H4A···S5^iv^	0.88	2.64	3.500 (6)	167.
N6A—H6A···S5	0.88	2.61	3.476 (6)	167.

Symmetry codes: (i) *x*, *y*, *z*−1; (ii) *x*, *y*−1, *z*; (iii) *x*, *y*, *z*+1; (iv) *x*, *y*+1, *z*.
